# White Matter Integrity Differences in 2‐Year‐Old Children Treated With ECMO: A Diffusion‐Weighted Imaging Study

**DOI:** 10.1111/ejn.70026

**Published:** 2025-02-17

**Authors:** Michaela Ruttorf, Julia Filip, Thomas Schaible, Meike Weis, Frank G. Zöllner

**Affiliations:** ^1^ Computer Assisted Clinical Medicine, Medical Faculty Mannheim Heidelberg University Mannheim Germany; ^2^ Mannheim Institute for Intelligent Systems in Medicine, Medical Faculty Mannheim Heidelberg University Mannheim Germany; ^3^ Department of Neonatology University Medical Centre Mannheim Mannheim Germany; ^4^ Department of Radiology and Nuclear Medicine, University Medical Centre Mannheim, Medical Faculty Mannheim Heidelberg University Mannheim Germany

**Keywords:** congenital diaphragmatic hernia, neurodevelopmental delays, neuroimaging, tract‐based spatial statistics

## Abstract

School‐aged and adolescent survivors of neonatal extracorporeal membrane oxygenation (ECMO) treatment still suffer from neurodevelopmental delays such as verbal, visuo‐spatial and working memory problems, motor dysfunction and sensorineural hearing loss, respectively, later in life, which is well‐documented by neuropsychological testing within follow‐up programs. In this study, we demonstrate that diffusion‐weighted imaging (DWI) in 2‐year‐old survivors of neonatal ECMO treatment reveals white matter (WM) alterations in brain regions related to neurodevelopmental outcome seen later in life. From the DWI data of 56 children, fractional anisotropy (FA), first fibre partial volume fraction estimate (F1), radial diffusivity (RD) and mean diffusivity (MD) are calculated and compared using tract‐based spatial statistics adapted to a paediatric brain atlas. Significant differences in FA, F1, RD and MD between the no‐ECMO and ECMO groups are seen in major WM tracts. Additionally, we examine individual diffusion measures by looking at 50 regions supplied with the paediatric brain atlas. We find the following regions to have significantly different means in the no‐ECMO compared with the ECMO group matching reports of neuropsychological delays found in behavioural tests: left anterior corona radiata, left anterior limb of internal capsule, left anterior commissure, left and right corpus callosum (genu, body and splenium), left and right crus of fornix and left tapetum. Analysing diffusion measures at an early stage of life serves as a good tool to detect structural WM changes in survivors of neonatal ECMO treatment. Compared with neuropsychological testing, DWI does not depend on the child's active participation.

## Graphical abstract

Statistically significant differences in the contrast no‐ECMO > ECMO are shown for fractional anisotropy (FA—red) and first fibre partial volume fraction estimate (F1—yellow) and in the contrast ECMO > no‐ECMO for radial diffusivity (RD—blue), respectively. The mean FA skeleton (green) is overlaid on the FA image.

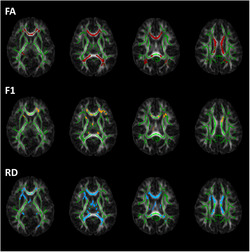

**Abbreviations**
CDH: congenital diaphragmatic hernia
DTI: diffusion tensor imaging
DWI: diffusion‐weighted imaging
ECMO: extracorporeal membrane oxygenation
F1: first fibre partial volume fraction estimate
FA: fractional anisotropy
MRI: magnetic resonance imaging
MD: mean diffusivity
RD: radial diffusivity
rCCA: right common carotid artery
WM: white matter

## 1 Introduction

Extracorporeal membrane oxygenation (ECMO) is a life‐saving technology for critically ill patients suffering from severe respiratory and/or cardiac failure, applied when maximal conventional therapy has failed. In 1975, neonatal ECMO was—for the first time— successfully administered by Bartlett and colleagues while treating a 1‐day‐old neonate after failing conventional therapies (Bartlett 2017). According to the 2022 Extracorporeal Life Support Organization (ELSO) registry report (The Extracorporeal Life Support Organization (ELSO) 2022), ECMO treatments are distributed across adult (54.5%), paediatric (18.9%) and neonatal (26.6%) patient groups—resulting in over 45,000 neonates with ECMO applied worldwide from 1989 to 2022. The majority of these neonates (73.0%) suffered from pulmonary problems, mostly from congenital diaphragmatic hernia (CDH), which occurs in approximately 1 out of 3500–4000 live births (Politis et al. 2021). A defect of the diaphragm leads to herniation of abdominal organs into the thoracic cavity impacting the growth and development of the lungs so that they remain small and underdeveloped. With the implementation of ECMO treatment, the survival rates after CDH repair—even in neonates with severe CDH—were improved (UK Collaborative ECMO Trail Group 1996). Despite technological enhancements and increasing clinical practice, significant morbidities among ECMO survivors with or without CDH—besides pulmonary problems—are neurologic complications, with the former showing even more neurodevelopmental problems during childhood (Danzer et al. 2013; Jaillard et al. 2003; Ijsselstijn and van Heijst 2014). In this study, we combined CDH and ECMO because it is not possible to investigate the effect of ECMO treatment in neonates only, that is, without underlying disease.

In the neonatal age group, neuronal plasticity is still very dynamic: Cortex and basal ganglia develop rapidly; central motor pathways are shaped establishing new cortico‐thalamic connections, as well as eliminating old ones (Johnston, Nakajima, and Hagberg 2002). A severe respiratory failure at this time in life interrupts these processes and affects developmental plasticity by modifying not only neurotransmission but also cellular signalling and neural connectivity/function, leading to regrowth of axons innervating the wrong targets. Although it was shown that neonatal ECMO survivors have an overall average and stable IQ from 2 to 5 to 8 years of age (Schiller et al. 2016; The Extracorporeal Life Support Organization (ELSO) n.d.), in many follow‐up programs, intelligence remains the primary outcome measure in these children (The Extracorporeal Life Support Organization (ELSO) n.d.). But only 5% to 10% of neonatal ECMO survivors suffer from severe neurologic complications, about 90% of ECMO survivors are at risk for subtler long‐term neurodevelopmental problems (Schiller et al. 2016; Madderom et al. 2016). Among the neurodevelopmental delays reported for CDH survivors with or without ECMO treatment are verbal, visuo‐spatial and working memory problems (Madderom et al. 2016), motor dysfunction and sensorineural hearing loss (McNally et al. 2006; Grover, Rintoul, and Hedrick 2018). But it was also shown that no‐ECMO treated CDH children have a better prognosis and are less likely to experience comorbidities that impact on long‐term outcome than their ECMO counterparts (Grover, Rintoul, and Hedrick 2018; Danzer et al. 2015). On the other hand, a lot of studies that looked at clinical parameters and their correlation to neurodevelopmental outcome later in life did not find any significant associations: neither in CDH survivors nor in CDH survivors treated with ECMO (see for example Madderom et al. 2016; Danzer et al. 2018; Kim et al. 2021; Friedman et al. 2008).

Based on these results, we decided to look only at differences in brain structure. To determine neurodevelopmental delay in ECMO survivors, usually, only neuropsychological assessment is used. However, it would be beneficial to identify children who are at higher risk for neurodevelopmental delay later in life as early as possible relying on information extracted directly from the brain. van den Bosch et al. (2015) found cortical thickness and global brain volumes in 8‐ to 15‐year‐old neonatal ECMO survivors, despite verbal memory problems, to be similar to healthy controls. They suppose that ECMO survivors suffer from very specific or subtle brain injuries that may not be identifiable using only high‐resolution structural magnetic resonance imaging (MRI). Therefore, we used diffusion‐weighted imaging (DWI) to detect alterations in microstructural characteristics in brain white matter (WM) such as WM integrity. WM integrity is undergoing rapid development in the neonatal period due to the dynamic plasticity, so CDH survivors with or without ECMO treatment may show different WM alterations. Moreover, WM integrity has been associated with neuropsychological outcome (Halliday et al. 2019) and behaviour (Kanai and Rees 2011).

This study aims to assess whether ECMO treatment associates with WM alterations in 2‐year‐old CDH survivors and whether these are relatable to the long‐term neuropsychological deficits observed in these children later in life. Up‐to‐now, there are only four studies investigating ECMO survivors by means of DWI: one compared normal age‐matched neonates with survivors of hypoxic–ischemic encephalopathy and ECMO (Seo, Kim, and Choi 2017), one looked at CDH survivors with or without ECMO at school‐age (Schiller et al. 2017a) and the other two looked at ECMO survivors at school‐age (Schiller et al. 2017b, 2019)—all with limited number of brain regions analysed. We decided to focus on 2‐year‐olds when lung growth is already in the middle of the alveolar stage and the recovery process from ECMO treatment no longer influences DTI parameter measurement. On the other hand, at 2 years of age, the children are not yet too old to benefit from additional therapies or exercises. Therefore, examining WM integrity in this age cohort may reveal more clearly side effects of ECMO treatment in the long run. We compare whole‐brain WM integrity extracted from DWI in CDH survivors after ECMO treatment to a no‐ECMO CDH control group. Then, we analyse 50 distinct brain regions including major WM tracts and subcortical WM in gyri. We expect to find WM alterations in ECMO survivors, specifically in areas associated with working memory, attention and motor function.

## 2 Materials and Methods

### 2.1 Patients

This study incorporates 56 children (mean age at measurement: 25.88 months, SD = 5.52 months) suffering from CDH, which were investigated in our institution according to the local follow‐up program including MRI (Zöllner et al. 2012; Henzler et al. 2017). All children born between 2011 and 2017 in whom the conventional MRI and DWI measurements were of diagnostic quality were considered for inclusion. The children were delivered at our institution with the antenatal diagnosis of CDH. Because of respiratory failure, 18 (seven girls, 11 boys) out of the 56 children received ECMO treatment (mean duration of ECMO: 9.41 days, SD = 3.18 days). ECMO treatment was initiated according to the recommendations suggested by the CDH EURO Consortium Consensus and ELSO (Snoek et al. 2016). Within the ECMO group, nine out of 18 children (four girls, five boys) received surgical reconstruction of the right common carotid artery (rCCA), the other nine children (three girls, six boys) received ligation. Thirty‐eight children (14 girls, 24 boys) served as the ‘control group’ suffering from CDH, but without ECMO treatment applied. As extensively described elsewhere, all children receiving ECMO were treated according to our postnatal management schedule (Buesing et al. 2007; Schaible 2015). In the MRI sessions, an additional diffusion‐weighted measurement—designed especially for the study purpose—was included. The children who received ECMO treatment stayed longer in hospital (mean duration: 75.18 days; SD = 38.00 days) than those without ECMO treatment (mean duration: 33.39 days, SD = 23.23 days). Written informed consent was obtained by the children's parents. The study was reviewed and approved by Ethikkommission II der Universität Heidelberg, Medizinische Fakultät, with the approval number: 2017‐595N‐MA, dated 29th January 2019; all research was performed in accordance with the Declaration of Helsinki.

### 2.2 DWI Data Acquisition

During DWI measurement, all children were sedated by intravenous administration of Propofol and continuously monitored by an anaesthesiologist. The measurement was performed on a 3‐T whole‐body MRI scanner (Magnetom TimTrio, Siemens Healthineers, Erlangen, Germany) using a 12‐channel head coil. For acquisition of DWI, a spin‐echo echo planar imaging sequence (TR = 8400 ms, TE = 84 ms, FoV = 192 × 192 mm^2^, BW = 1930 Hz/px) was used. Forty‐seven slices (voxel size = 2.0 mm^3^, no gap) were acquired in interleaved slice order using GRAPPA acceleration factor 2 and two averages. Diffusion weighting was performed in multidirectional diffusion weighting mode along 30 noncollinear directions with *b* = 1000 s/mm^2^. Additionally, a single non‐diffusion weighted volume (*b* = 0 s/mm^2^) was acquired with every average. Acquisition time was 5:58 min.

### 2.3 DWI Data Processing and Statistics

For DWI analyses, data were denoised first using MRtrix3 (Tournier et al. 2019) (*dwidenoise*) and then preprocessed using the FMRIB software library v 6.0.2 (Jenkinson et al. 2012) running on Ubuntu 18.04.3 LTS. After correction of eddy current distortions (*eddy*) and subject movement, we used *bet* routine with centre‐of‐gravity (−c) option to extract brain masks and adjusted fractional intensity threshold (*f* = 0.2) and vertical gradient in fractional intensity threshold (*g* = −0.1) option, accordingly (Popescu et al. 2012). To calculate fractional anisotropy (FA) from eigenvector maps, *dtifit* routine with weighted least‐squares regression was used. From these results, radial diffusivity (RD) values were determined. A modified version of Tract‐Based Spatial Statistics (TBSS) (Smith et al. 2006) was used for voxelwise statistical analyses. All scripts were changed as to register all children's FA data onto the Johns Hopkins University MRI/DTI Pediatric Brain Atlas for 2‐year‐olds (https://cmrm.med.jhmi.edu/) (Oishi et al. 2013) in a first step; afterwards, a mean FA image was created and thinned to a mean FA skeleton, which represents the centres of all tracts common to the group. The projection of every child's aligned FA data onto the mean FA skeleton was fed into voxelwise cross‐subject statistics (Winkler et al. 2014) with 5000 permutations of the data using age and sex as covariates of no interest. For analyses of RD and MD data, we used a TBSS script (*tbss_non_FA*) adapted for 2‐year‐olds by changing all references to standard space template images and masks. Furthermore, crossing‐fibre parameters were estimated using *bedpostX* routine with Rician noise option and three fibres modelled per voxel. The resulting fibre orientation and partial volume fraction estimates were analysed using a TBSS script (*tbss_x*) (Jbabdi, Behrens, and Smith 2010) for crossing fibres adapted for 2‐year‐olds by changing all references to standard space template images and masks. All resulting projections were, again, fed into whole‐brain voxelwise cross‐subject statistics using age and sex as covariates of no interest. All TBSS analysis results were acquired using threshold‐free cluster enhancement (Smith and Nichols 2009) and are fully corrected for multiple comparisons using family‐wise error (FWE) rate.

For statistical correlation analyses, mean values of diffusion measures were extracted from the corresponding parameter maps using masks from the Johns Hopkins University Pediatric Brain Atlas parcellation file. The masks were chosen according to the Johns Hopkins University ICBM‐DTI‐81 WM atlas (Mori et al. 2008) for adults included in FSL (for more details about masks selected see Table 1). We did this to increase comparability to later studies when our children were older or to studies that include only older children/adolescents. Although much of brain development occurs during the first few years of life, this process continues well beyond infancy (Lebel et al. 2008) and throughout adolescence.

**TABLE 1:** Labels of masks used in analyses and corresponding names of brain regions. The masks were selected from the Lookup Table of the Johns Hopkins University MRI/DTI Pediatric Brain Atlas for 2‐year‐olds corresponding to masks used in ICBM‐DTI‐81 WM atlas. R/L denotes right/left hemisphere, respectively.

Labels		Region
ICBM‐DTI‐81	JHU_pediatric24_SS	
ant_CC	Genu_CC_R	Genu of corpus callosum R/L
	Genu_CC_L	
CC	Body_CC_R	Body of corpus callosum R/L
	Body_CC_L	
post_CC	Splenium_CC_R	Splenium of corpus callosum R/L
	Splenium_CC_L	
R_cer_peduncle	CP_R	Cerebral peduncle R
L_cer_peduncle	CP_L	Cerebral peduncle L
R_alic	ALIC_R	Anterior limb of internal capsule R
L_alic	ALIC_L	Anterior limb of internal capsule L
R_plic	PLIC_R	Posterior limb of internal capsule R
L_plic	PLIC_L	Posterior limb of internal capsule L
R_Ant_cor_rad	ACR_R	Anterior corona radiata R
L_Ant_cor_rad	ACR_L	Anterior corona radiata L
R_Sup_cor_rad	SCR_R	Superior corona radiata R
L_Sup_cor_rad	SCR_L	Superior corona radiata L
R_Post_cor_rad	PCR_R	Posterior corona radiata R
L_Post_cor_rad	PCR_L	Posterior corona radiata L
R_ilf	SS_R	Sagittal stratum (include inferior longitudinal fasciculus and inferior fronto‐occipital fasciculus) R
L_ilf	SS_L	Sagittal stratum (include inferior longitudinal fasciculus and inferior fronto‐occipital fasciculus) L
R_unc	EC_R	External capsule R
L_unc	EC_L	External capsule L
R_cing	CGC_R	Cingulum (cingulate gyrus) R
L_cing	CGC_L	Cingulum (cingulate gyrus) L
R_cing_hipp	CGH_R	Cingulum (hippocampus) R
R_cing_hipp	CGH_L	Cingulum (hippocampus) L
R_hipp	Fx/ST_R	Fornix (cres)/stria terminalis (cannot be resolved with current resolution) R
L_hipp	Fx/ST_L	Fornix (cres)/stria terminalis (cannot be resolved with current resolution) L
R_slf	SLF_R	Superior longitudinal fasciculus R
L_slf	SLF_L	Superior longitudinal fasciculus L
R_unc_needsTBC	UNC_R	Uncinate fasciculus R
L_unc_needsTBC	UNC_L	Uncinate fasciculus L
R_tapetum	Tapetum_R	Tapetum R
L_tapetum	Tapetum_L	Tapetum L
	AnteriorComm_L	Anterior commissure L
	AnteriorComm_R	Anterior commissure R
	AnsaLenticularis_L	Ansa lenticularis L
	AnsaLenticularis_R	Ansa lenticularis R
	OpticTract_L	Optic tract L
	OpticTract_R	Optic tract R
	LenticularFasc_L	Lenticular fasciculus L
	LenticularFasc_R	Lenticular fasciculus R
	SubstriatumWM_L	Sub‐striatum white matter L
	SubstriatumWM_R	Sub‐striatum white matter R
	ML_L	Medial lemniscus L
	ML_R	Medial lemniscus R
	SFO_L	Superior fronto‐occipital fasciculus L
	SFO_R	Superior fronto‐occipital fasciculus R
	IFO_L	Inferior fronto‐occipital fasciculus L
	IFO_R	Inferior fronto‐occipital fasciculus R
	PTR_L	Posterior thalamic radiation (include optic radiation) L
	PTR_R	Posterior thalamic radiation (include optic radiation) R

We refrained from performing tractography considering the still persisting problems with the tracking algorithms—even in phantoms—as described by Schilling et al. (2019). Furthermore, the atlas‐based approach we used here offers a better predictive accuracy than approaches including streamline tractography (Ressel et al. 2018).

For testing of categorical and continuous variables, we performed a *χ* ^2^ test with *α* = 0.05 and Welch's *t*‐tests with *α* = 0.05, respectively, as implemented in MATLAB R2013a (The MathWorks Inc., Natick, MA, USA). For further analyses of FA, RD and MD values taken from each individual mask, we tested for normality in each sample, using the Shapiro–Wilk test (Shapiro and Wilk 1965) with *α* = 0.05 and concluding that normality could not be assumed. Using the Levene’s (1960) test with *α* = 0.05, we tested whether variances could be assumed equal for both groups. We concluded that this was not the case. Therefore, the nonparametric Brunner and Munzel’s (2000) test with *α* = 0.025 corrected for multiple inference (Bonferroni correction) was chosen for further analysis. This test is specifically designed to compare the location of two samples in the possible presence of unequal variances. It does not assume normality (Hays et al. 2013).

## 3 Results

Due to excessive head motion (> 2 mm in translation and 2° in rotation) or application of wrong measurement protocols, seven children—one from the ECMO group and six from the no‐ECMO group—were excluded. The sample size for further analyses is now 49.

All structural MRI measurements were checked for clinical evidence by an experienced paediatric radiologist to exclude those children with obvious brain lesions. Because we have limited clinical information of the children due to lack of a digital patient management system, additional data—including data on neurodevelopmental assessment—is not available. In the two groups, the clinical parameters relevant for decision of ECMO treatment (see guidelines (Snoek et al. 2016) for details) are significantly different because otherwise the children in the ECMO group would not have needed ECMO.

### 3.1 Statistical Tests

We performed Welch's *t*‐tests on age and duration of stay in hospital. There is no significant difference between the ECMO group and the no‐ECMO group in terms of age (*t*[28.262] = 1.523, *p* = 0.139). There is a significant difference between the ECMO group and the no‐ECMO group in terms of duration of stay in hospital (*t*[22.526] = 4.146, *p* < 0.001), which is caused by the need for ECMO treatment. We calculated Spearman's Rho to investigate possible correlations between diffusion measures (FA, RD and MD) and duration of stay in hospital within the ECMO group. After correction for multiple comparison, we found no significant correlation of DTI measures with duration of stay in hospital.

Furthermore, we performed a *χ* ^2^ test on sex. There is no significant difference between the ECMO group and the no‐ECMO group in terms of sex (*χ* ^2^(1, *N* = 49) = 0.030, *p* = 0.862).

### 3.2 Analyses of Diffusion Measures

Within the ECMO group, there is no significant difference in diffusion measures between children receiving ligation and those receiving surgical reconstruction of rCCA. For further analyses, we merged the two ECMO subgroups.

#### 3.2.1 TBSS Analyses

**FIGURE 1:**
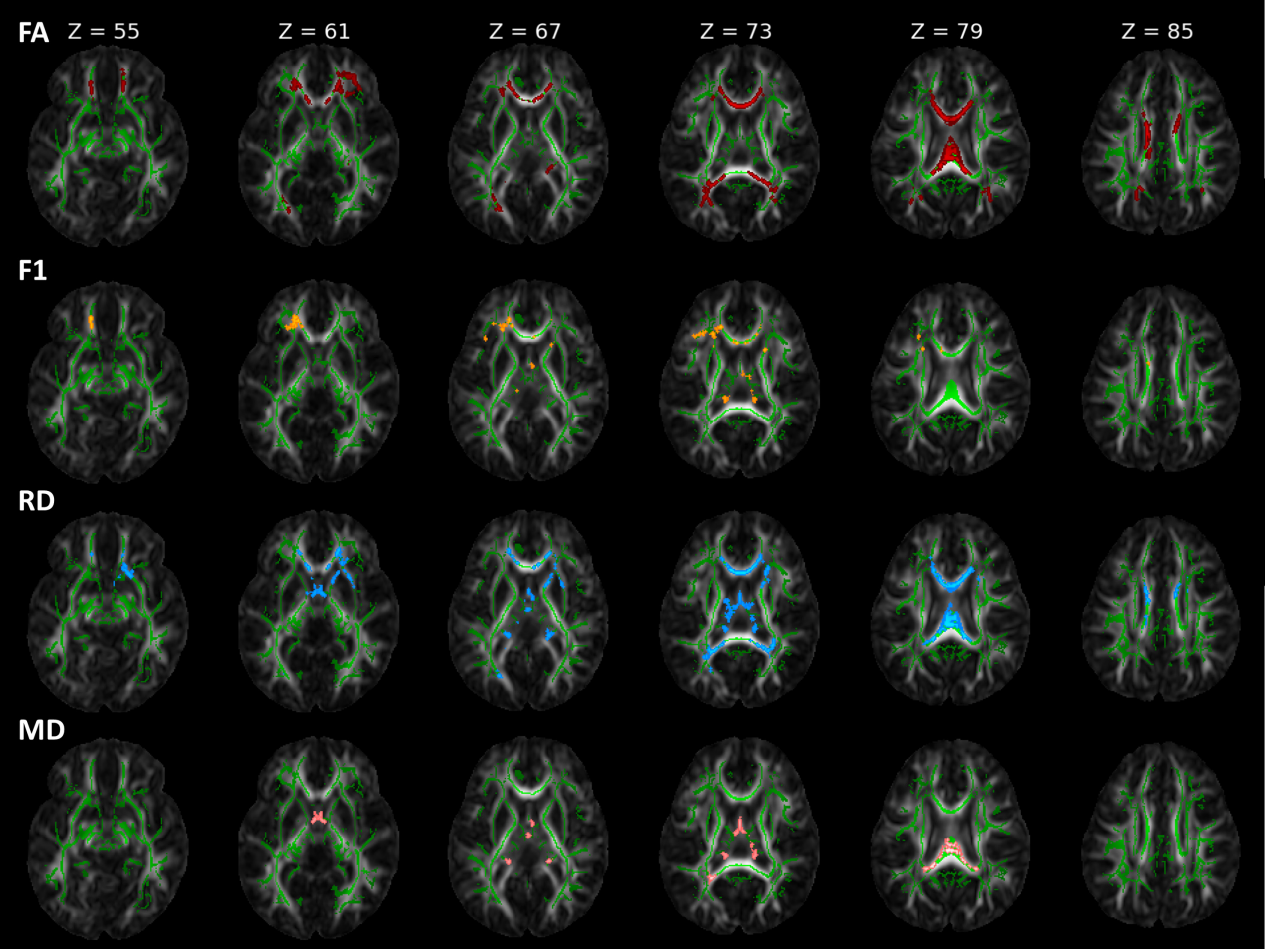
Voxelwise statistical analysis results of fractional anisotropy (FA), first fibre partial volume fraction estimate (F1), radial diffusivity (RD) and mean diffusivity (MD). The mean FA skeleton (green) is overlaid on the FA image. Statistically significant differences in the contrast no‐ECMO > ECMO are shown for FA (red) and F1 (yellow). Statistically significant differences in the contrast ECMO > no‐ECMO are shown for RD (blue) and MD (pink). The slices are displayed in neurological orientation.

In the whole‐brain voxelwise analysis, we find significantly higher (*p*_FWE_ < 0.05) FA values in the no‐ECMO group compared with the ECMO group (Figure 1, first panel) in centres of the following WM tracts: left and right anterior corona radiata, left and right corpus callosum (genu, body and splenium), left and right posterior thalamic radiation, left superior corona radiata, left and right middle occipital gyrus, right crus of fornix, left precuneus, left superior parietal gyrus and left superior occipital gyrus, as well as right inferior frontal gyurs and right middle frontal gyrus.

Because FA changes in crossing fibre regions are difficult to interpret, we additionally performed a crossing fibre analysis setting three fibres per voxel. In the whole‐brain voxelwise analysis, we find significantly higher (*p*_FWE_ < 0.05) first fibre partial volume fraction estimates (F1—Figure 1, second panel) in the no‐ECMO group compared with the ECMO group in the centres of the following WM tracts: left and right anterior corona radiata, left and right corpus callosum (genu, body and splenium), left and right superior corona radiata, right crus of fornix, left posterior thalamic radiation, right anterior limb of internal capsule, left external capsule, left cingulum (cingulate gyrus), left and right thalamus, left and right midbrain, left inferior frontal gyrus and right superior frontal gyrus.

There are 11 WM tracts with higher FA and higher F1 values in the no‐ECMO group compared with the ECMO group. In these brain regions, higher F1 values indicate higher orientation of fibres and fibre coherence. To support this finding, we further analysed RD and MD values, which give insight into radial and mean diffusivity. A whole‐brain voxelwise analysis revealed significantly higher (*p*_FWE_ < 0.05) RD values in the ECMO group compared with the no‐ECMO group in the centres of the following WM tracts: right anterior corona radiata, left and right corpus callosum (genu, body and splenium), left and right anterior commissure, left posterior thalamic radiation, left crus of fornix, left and right thalamus and left superior corona radiata (Figure 1, third panel). For MD, a whole‐brain voxelwise analysis revealed significantly higher (pFWE < 0.05) values in the ECMO group compared with the no‐ECMO group in the centres of the following WM tracts: left and right corpus callosum (body and splenium), left and right anterior commissure, left posterior thalamic radiation and left crus of fornix (Figure 1, fourth panel).

In total, there are five WM tracts with higher FA, higher F1, lower RD and lower MD values in the no‐ECMO group compared with the ECMO group: left and right corpus callosum (body and splenium) and left posterior thalamic radiation.

#### 3.2.2 Atlas‐Based ROI Analyses

**FIGURE 2:**
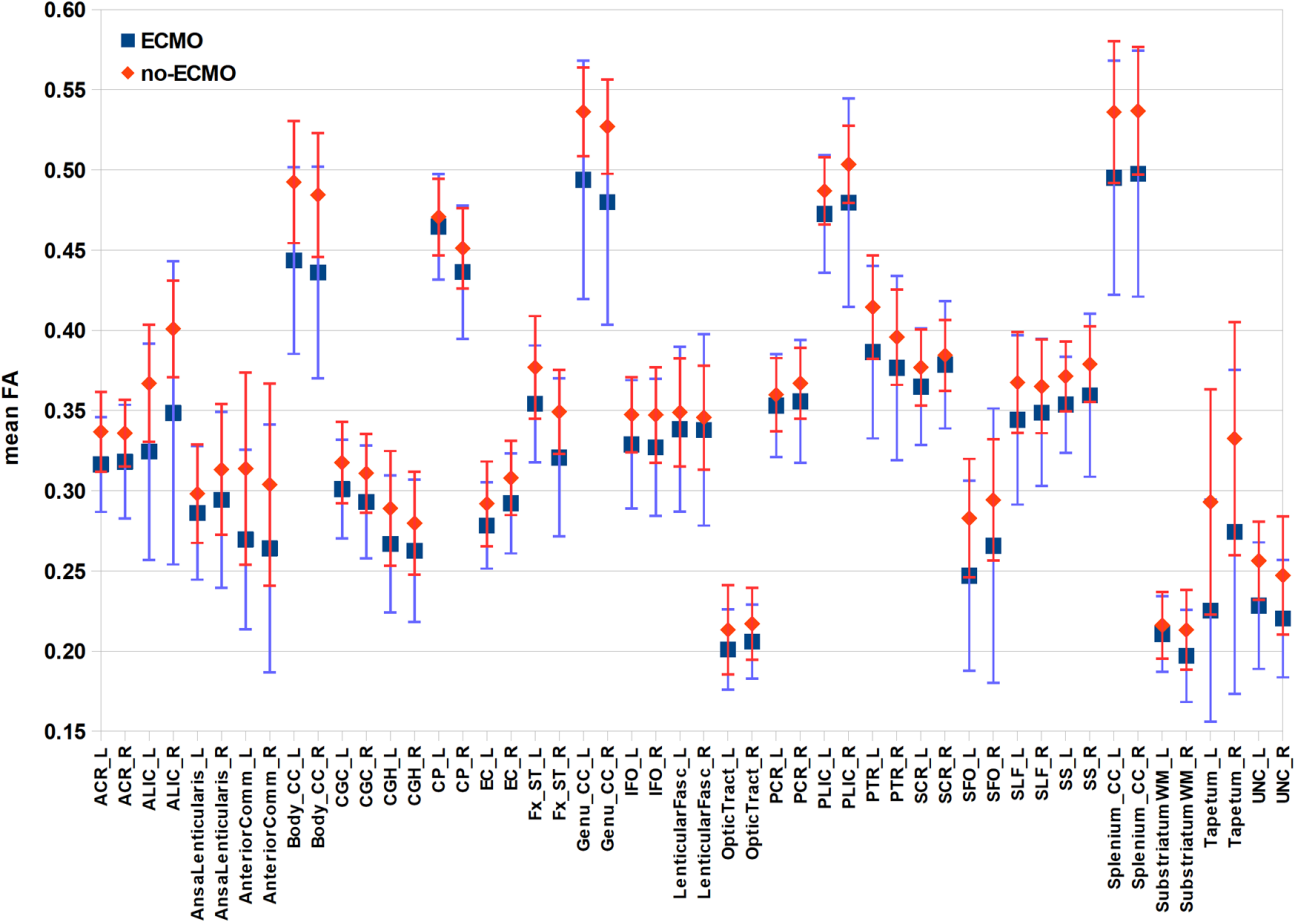
Mean fractional anisotropy (FA) values of white matter atlas masks. The mean FA values per mask and their standard deviations are plotted for both groups (ECMO, no‐ECMO). The masks were taken from the Johns Hopkins University MRI/DTI Pediatric Brain Atlas for 2‐year‐olds (see Table 1).

Because whole‐brain voxelwise statistical analyses compare the group means by projecting diffusion measures onto the centres of all WM tracts common to the group, we additionally performed atlas‐based analyses by looking at the individual FA, RD and MD values per mask. In Figure 2, the mean FA values per mask for both groups are shown. It is obvious that all mean FA values of the no‐ECMO group are higher than the corresponding mean FA values of the ECMO group. To statistically examine the distribution of mean FA values of the ECMO and no‐ECMO groups, we performed Brunner–Munzel tests.

We find a significant difference in no‐ECMO > ECMO in mean FA values in the following masks: left anterior corona radiata (W^BF^[33.355] = 2.717, *p* = 0.005), left and right anterior limb of internal capsule (left: W^BF^[34.366] = 3.744, *p* < 0.001; right: W^BF^[25.533] = 2.849, *p* = 0.004), left anterior commissure (W^BF^[35.507] = 2.291, *p* = 0.014), left and right genu of corpus callosum (left: W^BF^[25.046] = 3.183, *p* = 0.002; right: W^BF^[28.129] = 3.939, *p* < 0.001), left and right body of corpus callosum (left: W^BF^[29.803] = 4.210, *p* < 0.001; right: W^BF^[22.566] = 3.297, *p* = 0.002), left and right splenium of corpus callosum (left: W^BF^[42.466] = 3.772, *p* < 0.001; right: W^BF^[35.855] = 3.608, *p* < 0.001), right external capsule (W^BF^[38.863] = 2.047, *p* = 0.024), left and right crus of fornix (left: W^BF^[37.057] = 2.794, *p* = 0.005; right: W^BF^[23.449] = 2.467, *p* = 0.011), left tapetum (W^BF^[34.655] = 3.801, *p* < 0.001) and left and right uncinate fasciculus (left: W^BF^[40.772] = 3.014, *p* = 0.002; right: W^BF^[40.340] = 2.995, *p* = 0.002).

**FIGURE 3:**
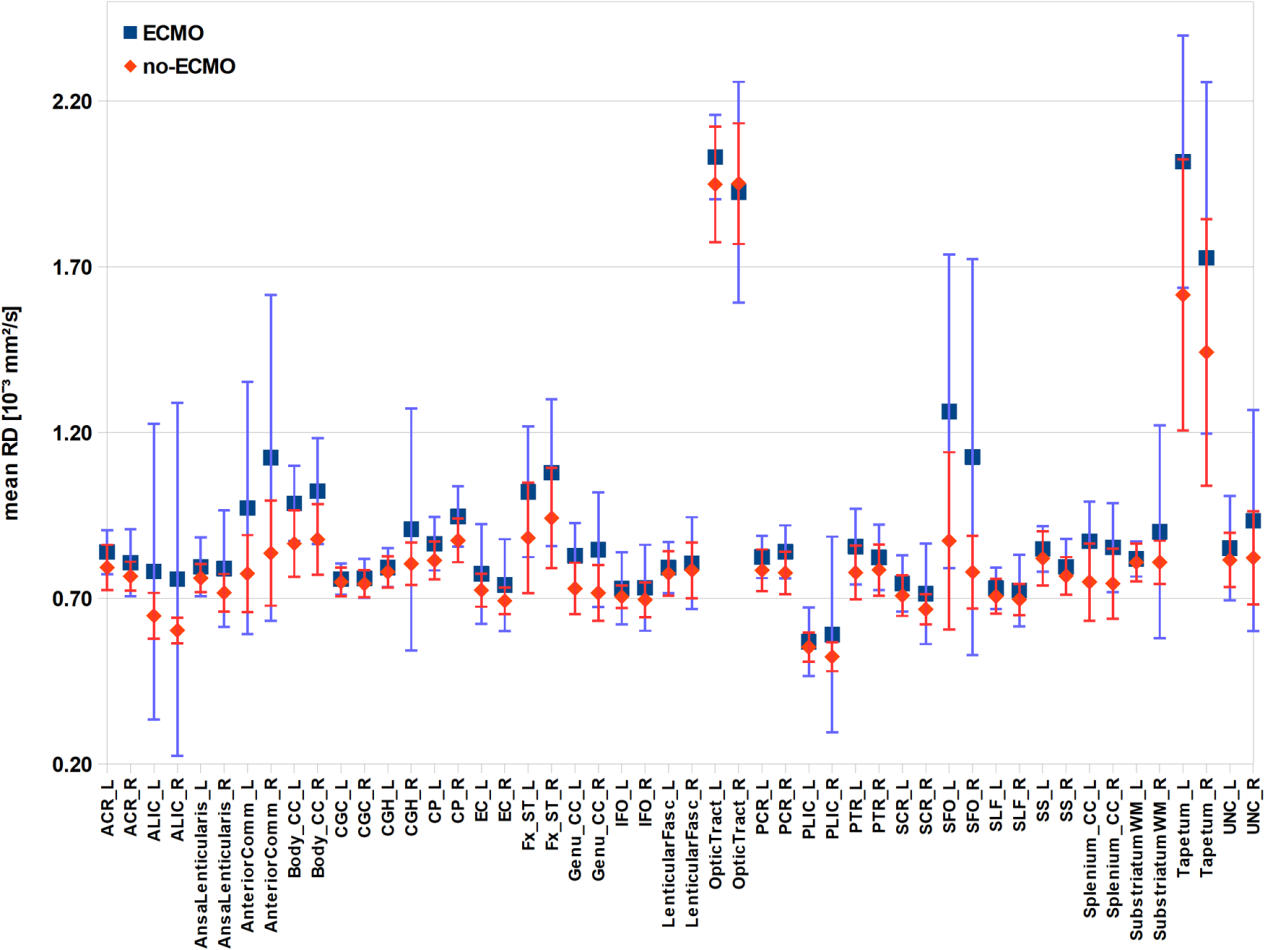
Mean radial diffusivity (RD) values of white matter atlas masks. The mean RD values of the masks and their standard deviations are plotted for both groups (ECMO, no‐ECMO). The masks were taken from the Johns Hopkins University MRI/DTI Pediatric Brain Atlas for 2‐year‐olds (see Table 1).

In Figure 3, the mean RD values per mask for both groups are shown. Almost all mean RD values of the ECMO group are higher than the corresponding mean RD values of the no‐ECMO group. Again, to statistically examine the distribution of mean RD values of the ECMO and no‐ECMO groups, we performed Brunner–Munzel test. In mean RD values, we find a significant difference in ECMO > no‐ECMO in the following masks: left anterior corona radiata (W^BF^[30.382] = −2.778, *p* = 0.005), left anterior limb of internal capsule (W^BF^[30.246] = −2.565, *p* = 0.008), right ansa lenticularis (W^BF^[32.436] = −2.696, *p* = 0.006), left and right anterior commissure (left: W^BF^[32.426] = −3.204, *p* = 0.002; right: WBF[29.027] = −3.306, *p* = 0.001), left and right cerebral peduncle (left: WBF[31.959] = −2.482, *p* = 0.009; right: W^BF^[26.033] = −3.323, *p* = 0.001), left and right crus of fornix (left: W^BF^[32.333] = −3.556, *p* = 0.001; right: W^BF^[26.643] = −2.881, *p* = 0.004), left and right genu of corpus callosum (left: W^BF^[34.521] = −4.563, *p* < 0.001; right: W^BF^[31.269] = −4.253, *p* < 0.001), left and right body of corpus callosum (left: W^BF^[37.409] = −4.701, *p* < 0.001; right: W^BF^[34.064] = −4.208, *p* < 0.001), left and right splenium of corpus callosum (left: W^BF^[46.098] = −5.691, *p* < 0.001; right: W^BF^[40.895] = −4.198, *p* < 0.001), left and right superior fronto‐occipital fasciculus (left: W^BF^[43.625] = −5.262, *p* < 0.001; right: W^BF^[25.313] = −2.971, *p* = 0.003), left and right posterior corona radiata (left: W^BF^[37.195] = −2.429, *p* = 0.010; right: W^BF^[29.115] = −3.220, *p* = 0.002), left optic tract (W^BF^[43.916] = −2.163, *p* = 0.018), left posterior thalamic radiation (W^BF^[28.883] = −2.850, *p* = 0.004), left tapetum (W^BF^[41.641] = −4.100, *p* < 0.001) and right uncinate fasciculus (W^BF^[42.510] = −2.601, *p* = 0.006).

**FIGURE 4:**
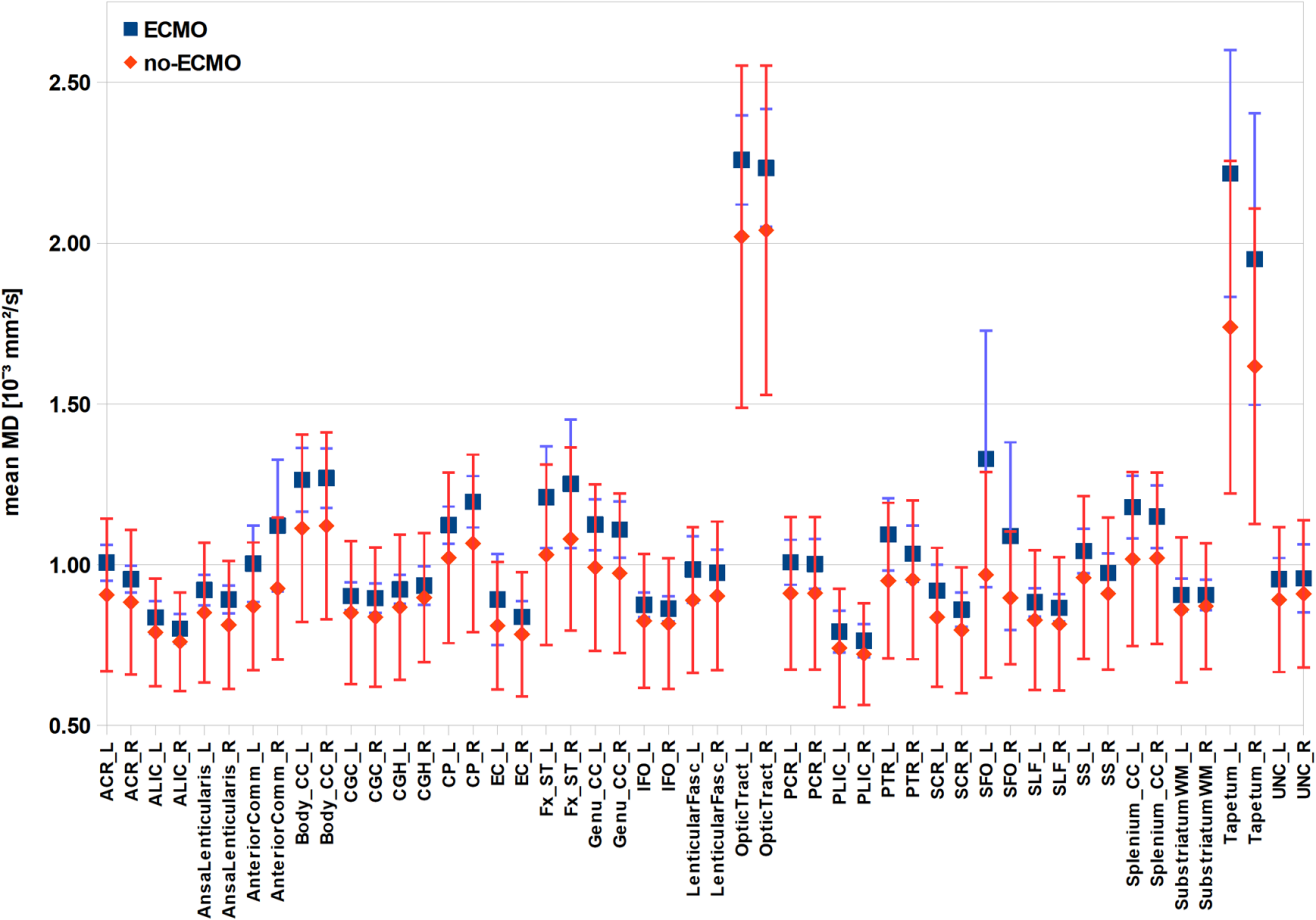
Mean mean diffusivity (MD) values of white matter atlas masks. The mean MD values of the masks and their standard deviations are plotted for both groups (ECMO, no‐ECMO). The masks were taken from the Johns Hopkins University MRI/DTI Pediatric Brain Atlas for 2‐year‐olds (see Table 1).

In Figure 4, the mean MD values per mask for both groups are shown. Almost all mean MD values of the ECMO group are higher than the corresponding mean MD values of the no‐ECMO group. Again, to statistically examine the distribution of mean MD values of the ECMO and no‐ECMO groups, we performed Brunner–Munzel test. In mean MD values, we find a significant difference in ECMO > no‐ECMO in the following masks: left anterior corona radiata (W^BF^[31.213] = −2.576, *p* = 0.008), left anterior limb of internal capsule (W^BF^[30.246] = −2.565, *p* = 0.008), right ansa lenticularis (W^BF^[36.173] = −3.595, *p* < 0.001), left and right anterior commissure (left: W^BF^[31.128] = −3.175, *p* = 0.002; right: WBF[31.565] = −3.891, *p* < 0.001), left and right cerebral peduncle (left: WBF[29.572] = −2.493, *p* = 0.009; right: W^BF^[23.973] = −2.782, *p* = 0.005), left and right crus of fornix (left: W^BF^[32.218] = −3.519, *p* < 0.001; right: W^BF^[26.269] = −2.547, *p* = 0.009), left and right genu of corpus callosum (left: W^BF^[32.418] = −3.778, *p* < 0.001; right: W^BF^[33.543] = −3.833, *p* < 0.001), left and right body of corpus callosum (left: W^BF^[38.463] = −3.583, *p* < 0.001; right: W^BF^[34.474] = −3.588, *p* < 0.001), left and right splenium of corpus callosum (left: W^BF^[45.918] = −5.947, *p* < 0.001; right: W^BF^(34.502) = −3.861, *p* < 0.001), left and right superior fronto‐occipital fasciculus (left: W^BF^[45.569] = −5.551, *p* < 0.001; right: W^BF^[27.659] = −3.416, *p* < 0.001), left and right posterior corona radiata (left: W^BF^[36.341] = −2.340, *p* = 0.013; right: W^BF^[33.049] = −3.420, *p* < 0.001), left optic tract (W^BF^[42.219] = −2.246, *p* = 0.015) and left tapetum (W^BF^[42.496] = −4.076, *p* < 0.001.

**FIGURE 5:**
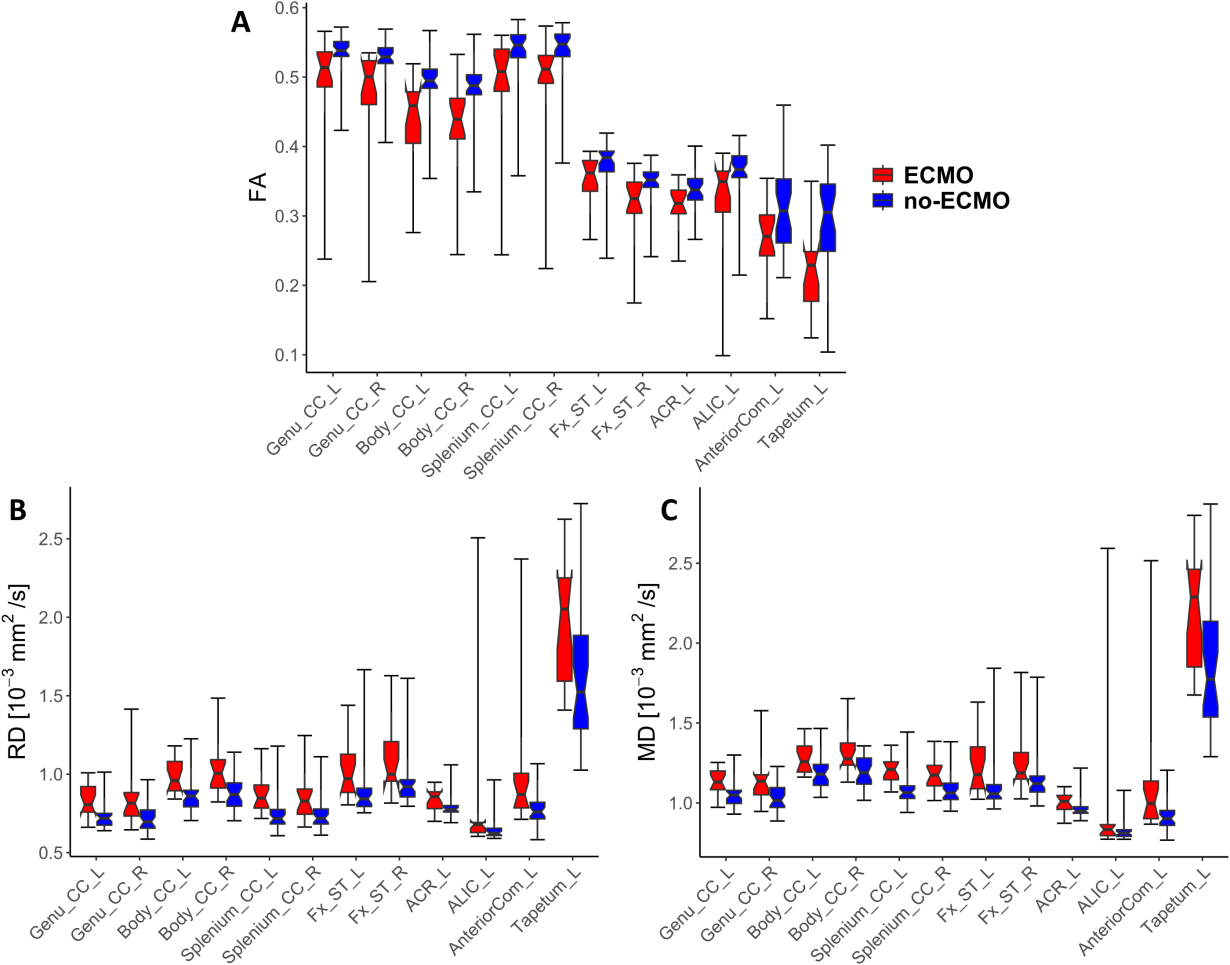
Significantly different fractional anisotropy (FA), radial diffusivity (RD) and mean diffusivity (MD) values of white matter atlas masks. (A) The FA values of the significantly different masks are plotted for both groups (ECMO, no‐ECMO). (B) The RD values of the significantly different masks are plotted for both groups (ECMO, no‐ECMO). (C) The MD values of the significantly different masks are plotted for both groups (ECMO, no‐ECMO). Centre lines show the group medians; notches correspond to 95% confidence interval of median; box limits indicate the 25th and 75th percentiles; whiskers extend to minimum and maximum values (for numerical data: see Table 2). The masks were taken from the Johns Hopkins University MRI/DTI Pediatric Brain Atlas for 2‐year‐olds (see Table 1).

In total, there are 12 brain masks with significantly higher FA and significantly lower RD and lower MD values in the no‐ECMO group compared with the ECMO group: left anterior corona radiata, left anterior limb of internal capsule, left anterior commissure, left and right corpus callosum (genu, body and splenium), left and right crus of fornix and left tapetum (see Figure 5 for a visual presentation).

The boxplots visualise very well the median and spread of values within each group; in all 12 brain masks, the medians of FA values in the no‐ECMO group are higher compared with the ECMO group (Figure 5A) and the spread of FA values is smaller. On the other hand, the medians of RD and MD values in the same regions are lower (Figure 5B,C) with the spread of RD/MD values again remaining smaller in the no‐ECMO group compared with the ECMO group. The numerical data of all medians and interquartile ranges are listed in Table 2.

**TABLE 2:** Median (Mdn) and interquartile range (IQR) values of fractional anisotropy (FA), radial diffusivity (RD) and mean diffusivity (MD) from brain regions shown in Figure 5 for both groups: ECMO and no‐ECMO. The masks were selected from the Lookup Table of the Johns Hopkins University MRI/DTI Pediatric Brain Atlas for 2‐year‐olds.

Name of atlas mask	Mdn FA (IQR)		Mdn RD (IQR) (10^−3^ mm^2^/s)		Mdn MD (IQR) (10^−3^ mm^2^/s)	
	ECMO	No‐ECMO	ECMO	No‐ECMO	ECMO	No‐ECMO
Genu_CC_L	0.51 (0.49–0.54)	0.54 (0.53–0.55)	0.81 (0.76–0.92)	0.72 (0.67–0.75)	1.13 (1.08–1.20)	1.05 (1.00–1.08)
Genu_CC_R	0.50 (0.46–0.52)	0.53 (0.52–0.54)	0.81 (0.73–0.88)	0.70 (0.65–0.78)	1.14 (1.05–1.18)	1.01 (0.97–1.10)
Body_CC_L	0.46 (0.40–0.48)	0.49 (0.48–0.51)	0.96 (0.89–1.08)	0.86 (0.79–0.90)	1.26 (1.19–1.36)	1.18 (1.11–1.24)
Body_CC_R	0.44 (0.41–0.47)	0.49 (0.47–0.50)	1.01 (0.91–1.09)	0.87 (0.79–0.95)	1.28 (1.24–1.38)	1.19 (1.11–1.28)
Splenium_CC_L	0.51 (0.48–0.54)	0.55 (0.53–0.56)	0.85 (0.78–0.93)	0.72 (0.68–0.78)	1.21 (1.15–1.26)	1.06 (1.02–1.11)
Splenium_CC_R	0.51 (0.49–0.53)	0.55 (0.53–0.56)	0.83 (0.74–0.91)	0.72 (0.68–0.78)	1.17 (1.11–1.24)	1.06 (1.02–1.12)
Fx_ST_L	0.36 (0.34–0.38)	0.38 (0.36–0.39)	0.97 (0.88–1.13)	0.84 (0.79–0.91)	1.18 (1.09–1.35)	1.06 (1.03–1.12)
Fx_ST_R	0.32 (0.30–0.35)	0.35 (0.34–0.36)	1.00 (0.96–1.21)	0.92 (0.85–0.97)	1.19 (1.15–1.32)	1.12 (1.06–1.17)
ACR_L	0.32 (0.30–0.34)	0.34 (0.32–0.35)	0.86 (0.78–0.89)	0.78 (0.76–0.80)	1.01 (0.96–1.05)	0.95 (0.94–0.98)
ALIC_L	0.35 (0.31–0.36)	0.37 (0.35–0.39)	0.68 (0.63–0.69)	0.62 (0.61–0.66)	0.83 (0.80–0.86)	0.81 (0.79–0.83)
AnteriorCom_L	0.27 (0.24–0.30)	0.31 (0.26–0.36)	0.87 (0.79–1.01)	0.76 (0.71–0.83)	1.00 (0.90–1.14)	0.9 (0.86–0.95)
Tapetum_L	0.23 (0.18–0.25)	0.31 (0.25–0.35)	2.05 (1.59–2.25)	1.52 (1.28–1.89)	2.29 (1.85–2.46)	1.77 (1.53–2.14)

## 4 Discussion

We could show that ECMO treatment associates with WM integrity in 2‐year‐old CDH survivors. DTI measures like FA include effects from many underlying parameters and are thus nonspecific. There are many physiological processes that may cause changes in imaging parameters to occur during brain development. Possible microstructural contributions include myelination or changes in axonal density or axon coherence. Concurrent changes in tract volume and water content also influence various imaging parameters. In the whole‐brain voxelwise analyses and in almost all WM masks, we clearly see higher FA values (see Figure 1, first panel, and Figure 2) and lower RD values (see Figure 1, third panel, and Figure 3) in the no‐ECMO group compared with the ECMO group. This may be due to various underlying parameters including axonal density, fibre orientation dispersion and degree of myelination. Roberts et al. (2005) and Friedrich et al. (2020) report no correlation between myelin content and FA. But there was a correlation between axon/fibre density and FA. This is also supported by Winston et al. (2014) who report a decrease in FA in focal cortical dysplasia in epilepsy patients due to an increase in extracellular space. Furthermore, Stikov et al. (2011) found that FA is most sensitive to the amount of extra‐axonal space, which is related to axon density as can be seen in areas with high directional coherence, such as the corpus callosum where FA increases. On the basis of these findings, increases in FA here seem to be merely based on either increase in axon density or axon coherence—when more axons are aligned along the same axis.

Our results show significantly lower FA values in the ECMO group in WM tracts associated with high axon coherence, such as the corpus callosum and the fornix. Besides, the first fibre partial volume fraction estimate F1 was lower in the ECMO group in these tracts supporting this conclusion. Simultaneously, RD and MD values in the same tracts are significantly higher indicating either less axon density or less axons aligned along the same axis. This suggests that, due to the very dynamic neuronal plasticity at the neonatal stage, children from the ECMO group experience neural connectivity, which leads to erroneous growth of axons that innervate the wrong targets. For a developing brain, exposure to hypoxic ischemic injury around the time of birth can be considered as a point of vulnerability in neurobiological development; the neurodevelopmental impairment ECMO survivors exhibit later in life match these findings. The most relevant brain regions (significant higher FA and significant lower RD and MD in the no‐ECMO group—Figure 5) we found in the individual FA, RD and MD values support the findings on impairment in neurodevelopmental outcome later in life reported by others (Madderom et al. 2016; McNally et al. 2006; Danzer et al. 2018; Kim et al. 2021; Friedman et al. 2008; Schiller et al. 2017b, 2019; Engle et al. 2017; Leeuwen et al. 2018; Madderom et al. 2013; Schiller and Tibboel 2019), which are (a) motor dysfunction (corpus callosum and anterior limb of internal capsule), (b) visuo‐spatial problems (corpus callosum and anterior commissure), (c) memory problems (crus of fornix) and (d) attention problems (anterior corona radiata and anterior commissure). The three parts of the corpus callosum (genu, body and splenium) connect the two cerebral hemispheres and are known to be involved in movement control, cognitive functions and vision. The anterior limb of the internal capsule relays motor and sensory information with ascending and descending fibres between the cerebral cortex and the pyramids of the medulla (Schünke et al. 2020). The crus of fornix acts as the major output tract of the hippocampus connecting it to various subcortical structures like the mammillary bodies and the anterior nucleus of thalamus. It is also a critical component of the Papez circuit, which is involved in learning and memory, emotion and social behaviour. The anterior corona radiata is part of the limbic‐thalamo‐cortical circuitry and consists of afferent and efferent fibres that connect the cerebral cortex and the brain stem. Both are involved in sensation and motor function, and the corona radiata connects motor and sensory nerve pathways between these structures. Niogi et al. (2010) provide evidence that microstructural integrity of the anterior corona radiata modulates executive attention. The anterior commissure plays a significant role in visual processing and memory, among others, as was shown by selectively inactivating the anterior commissure in monkeys (see review by Fenlon et al. 2021).

There was no significant difference in diffusion measures between ligation and rCCA within the ECMO group, which is in line with Duggan et al. (2015) who were able to show that there appear to be no significant differences in the incidence of brain lesions in patients who undergo carotid repair at time of decannulation from ECMO compared with those undergoing ligation. Likewise, clinical parameters such as persistent pulmonary hypertension, arterial blood gas values, preductal and postductal oxygen saturation and cardiovascular dysfunction are not correlated with adverse neurodevelopmental delay (Danzer et al. 2018; Church et al. 2018). Gestational age is not an important factor of developmental outcomes after ECMO as well (Kim et al. 2021). The study by Madderom et al. (2016) assessed the influence of medical variables on the neuropsychologic domains on which adolescent survivors of neonatal ECMO treatment showed impaired performance and did not find any of these variables to be a significant predictor. A fourth study identified ventilator time/need for ECMO as the only independent predictor of motor problems at age 1 (Friedman et al. 2008). Danzer et al. (2018) investigated the influence of need and timing of ECMO in relation to CDH repair on short‐term neurodevelopmental outcomes in children of 2 years of age. They did not find any association of duration of ECMO with a higher likelihood of adverse cognitive, language or motor outcome or increased risk of neuromuscular hypotonicity. Furthermore, if children who were in need of neonatal ECMO were repaired early or late in the ECMO course did not have an impact on their neurodevelopmental outcome.

The advantage of DWI lies in looking only at the neurobiology, for example WM integrity being independent of the subjective assessment of the person who performs the measurement. Neurodevelopmental assessment tools, which often rely on parent‐reported outcomes and the training of the provider completing the assessment, are challenging to implement in a young population (Cimbak and Buchmiller 2024). DWI, on the other hand, does not depend on the child's active participation. Compared with other DWI studies where only limited brain regions (six regions; Schiller et al. 2017a, 2017b, 2019 or nine regions; Seo, Kim, and Choi 2017) were selected, we performed whole‐brain TBSS analyses based on a paediatric atlas for 2‐year‐olds. Five WM tracts show a significant difference in all four diffusion measures (FA, F1, RD, MD). We then selected the corresponding atlas masks plus those equivalent to the masks given in the Johns Hopkins University ICBM‐DTI‐81 WM atlas for adults shipped with FSL for further analyses on individual level (see Table 1) and find significantly different group means in 12 masks that match the results of the neurodevelopmental assessments reported in the literature (see above).

## 5 Conclusion

Analysing diffusion measures such as FA, F1, RD and MD serves as a tool to detect early structural WM changes in survivors of neonatal ECMO treatment. The differences seem to lie in lacking axon coherence in fibre bundles that develop early in life. As these children are at risk for developing cognitive disorders during childhood that persist into adolescence (Madderom et al. 2016; McNally et al. 2006), we recommend focused screening to support therapeutic strategies. Schiller et al. (2019) have already shown that training‐induced changes in WM microstructure, for example after Cogmed Working‐Memory Training, are possible and associated with better verbal working‐memory. This procedure should also be applicable to the other areas of neurodevelopmental impairment seen later in life because, although much of brain development occurs during the first few years of life, this process continues well beyond infancy (Lebel et al. 2008) and throughout adolescence. Targeted training can therefore help to mitigate the neurodevelopmental deficits ECMO survivors face later in life.

## Author Contributions

**Michaela Ruttorf:** data curation, formal analysis, methodology, visualisation, writing – original draft, writing – review and editing. **Julia Filip:** data curation, validation. **Thomas Schaible:** resources, supervision. **Meike Weis:** conceptualisation, project administration, resources, supervision. **Frank Gerrit Zöllner:** data curation, funding acquisition, project administration, supervision, writing – review and editing.

## Conflicts of Interest

The authors declare no conflicts of interest.

### Peer Review

The peer review history for this article is available at https://www.webofscience.com/api/gateway/wos/peer‐review/10.1111/ejn.70026.

## Data Availability

The datasets supporting the conclusions of this article are available upon request from meike.weis@medma.uni-heidelberg.de.
